# *N*,*N*′-(Propane-1,3-di­yl)bis­(*p*-toluene­sulfonamide)

**DOI:** 10.1107/S1600536811030820

**Published:** 2011-08-17

**Authors:** Islam Ullah Khan, Tahir Ali Sheikh, Ejaz, William T. A. Harrison

**Affiliations:** a Materials Chemistry Laboratory, Department of Chemistry, GC University, Lahore 54000, Pakistan; b Department of Chemistry, University of Aberdeen, Meston Walk, Aberdeen AB24 3UE, Scotland

## Abstract

The complete mol­ecule of the title compound, C_17_H_22_N_2_O_4_S_2_, is generated by crystallographic twofold symmetry, with one C atom lying on the rotation axis. The dihedral angle between the benzene rings is 44.04 (7)° and the conformation of the central N—C—C—C group is *gauche*. In the crystal, mol­ecules are linked by N—H⋯O hydrogen bonds, generating corrugated (010) sheets, and weak C—H⋯O inter­actions consolidate the packing.

## Related literature

For the related structure of *N*,*N*′-ethyl­enebis(*p*-toluene­sulfonamide), see: Gajadhar-Plummer *et al.* (2001 ▶).
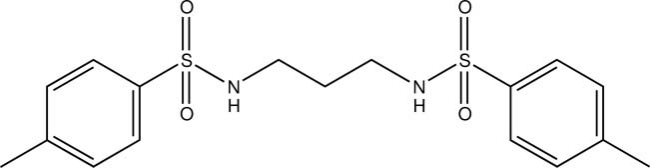


## Experimental

### 

#### Crystal data

C_17_H_22_N_2_O_4_S_2_
*M* *_r_* = 382.49
Orthorhombic, 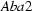

*a* = 12.3169 (9) Å
*b* = 18.0787 (15) Å
*c* = 8.3819 (5) Å
*V* = 1866.4 (2) Å^3^
*Z* = 4
Mo *K*α radiation
μ = 0.31 mm^−1^
*T* = 296 K
0.52 × 0.46 × 0.36 mm

#### Data collection

Bruker APEXII CCD diffractometer
Absorption correction: multi-scan (*SADABS*; Bruker, 2007 ▶) *T*_min_ = 0.856, *T*_max_ = 0.897
4996 measured reflections
1625 independent reflections
1472 reflections with *I* > 2σ(*I*)
*R*_int_ = 0.019

#### Refinement

*R*[*F* ^2^ > 2σ(*F* ^2^)] = 0.028
*wR*(*F* ^2^) = 0.078
*S* = 1.07
1625 reflections
119 parameters
1 restraint
H atoms treated by a mixture of independent and constrained refinement
Δρ_max_ = 0.21 e Å^−3^
Δρ_min_ = −0.20 e Å^−3^
Absolute structure: Flack (1983 ▶), 372 Friedel pairs
Flack parameter: 0.12 (11)

### 

Data collection: *APEX2* (Bruker, 2007 ▶); cell refinement: *SAINT* (Bruker, 2007 ▶); data reduction: *SAINT*; program(s) used to solve structure: *SHELXS97* (Sheldrick, 2008 ▶); program(s) used to refine structure: *SHELXL97* (Sheldrick, 2008 ▶); molecular graphics: *ORTEP-3* (Farrugia, 1997 ▶); software used to prepare material for publication: *SHELXL97* .

## Supplementary Material

Crystal structure: contains datablock(s) I, global. DOI: 10.1107/S1600536811030820/om2455sup1.cif

Structure factors: contains datablock(s) I. DOI: 10.1107/S1600536811030820/om2455Isup2.hkl

Supplementary material file. DOI: 10.1107/S1600536811030820/om2455Isup3.cml

Additional supplementary materials:  crystallographic information; 3D view; checkCIF report

## Figures and Tables

**Table 1:** Hydrogen-bond geometry (Å, °)

*D*—H⋯*A*	*D*—H	H⋯*A*	*D*⋯*A*	*D*—H⋯*A*
N1—H1⋯O2^i^	0.82 (3)	2.24 (3)	2.974 (2)	149 (2)
C7—H7*C*⋯O1^ii^	0.96	2.45	3.264 (3)	142

Symmetry codes: (i) 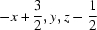; (ii) 
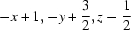
.

**Fig. 1.**
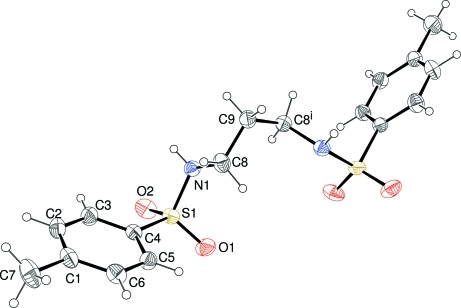
The molecular structure of (I) showing 30% displacement ellipsoids. Symmetry code: (i) 1–x, 1–y, z.

**Fig. 2.**
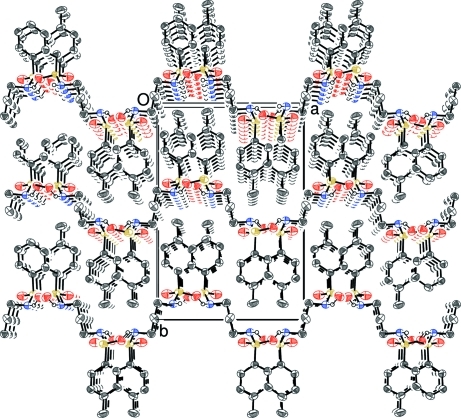
View approximately down [001] of the packing in (I) showing the interdigitated (010) sheets of molecules. All C-bound H atoms are omitted for clarity.

## supplementary crystallographic information

### Comment

As part of our ongoing structural studies of sulfonamides, the synthesis and structure of the title compound, (I), are now described.

The complete molecule of (I) is generated by crystallographic twofold symmetry (Fig. 1) with atom C9 lying on the rotation axis. The diehdral angle between the benzene rings is 44.04 (7)°. The conformation of the atoms of the central chain is *gauche* [N1—C8—C9—C8^i^ = 75.53 (14)°; (i) = 1–x, 1–y, *z*] whereas the torsion angle for S1—N1—C8—C9 of -163.87 (15)° indicates a near anti conformation for these atoms. The bond-angle sum for N1 of 341.7° seems to indicate an intermediate valence state between *sp*^2^ and *sp*^3^ hybridization (expected bond angle sums = 328.5 and 360°, respectively).

In the crystal, the molecules are linked by N—H···O hydrogen bonds (Table 1), to generate corrugated (010) sheets (Fig. 2). A weak C—H···O interaction may help to consolidate the packing. There is no aromatic π-π stacking in the crystal of (I).

The structure of the related compound *N*,*N*'-ethylenebis(*p*-toluenesulfonamide), (II), has been reported (Gajadhar-Plummer *et al.*, 2001), in which an ethlyene bridge links the *p*-toluenesulfonamide units compared to a propylene bridge in (I). The complete molcule of (II) is generated by crystallographic inversion symmetry, thus the central N—C—C—N bridge is constrained to have a perfect anti conformation. The S—N—C—C torsion angle of -98.0 (2)° in (II) is also quite different to the equivalent torsion angle in (I).

### Experimental

A mixture of 1,3-diaminopropane (0.0067 mol, 0.561 ml) and *p*-toluenesulfonyl chloride (0.0135 mol, 2.55 g) was stirred in 20 ml distilled water while maintaining the pH of the solution at about 9.0 with sodium carbonate solution (3%). The progress of the reaction was monitored by TLC: on completion, the white precipitate formed was filtered, washed with distilled water and dried. Colourless blocks of (I) were recrystallized from methanol.

### Refinement

The N-bound H atom was located in a difference map and its position was freely refined with *U*_iso_(H) = 1.2*U*_eq_(N). The C-bound hydrogen atoms were placed in calculated positions (C—H = 0.97–0.98 Å) and refined as riding atoms with *U*_iso_(H) = 1.2*U*_eq_(C) or 1.5*U*_eq_(methyl C). The methyl group was allowed to rotate, but not to tip, to best fit the electron density.

### Crystal data

**Table:**

C_17_H_22_N_2_O_4_S_2_	*F*(000) = 808
*M**_r_* = 382.49	*D*_x_ = 1.361 Mg m^−^^3^
Orthorhombic, *A**b**a*2	Mo *K*α radiation, λ = 0.71073 Å
Hall symbol: A 2 -2ac	Cell parameters from 2756 reflections
*a* = 12.3169 (9) Å	θ = 2.8–28.3°
*b* = 18.0787 (15) Å	µ = 0.31 mm^−^^1^
*c* = 8.3819 (5) Å	*T* = 296 K
*V* = 1866.4 (2) Å^3^	Block, colourless
*Z* = 4	0.52 × 0.46 × 0.36 mm

### Data collection

**Table:**

Bruker APEXII CCD diffractometer	1625 independent reflections
Radiation source: fine-focus sealed tube	1472 reflections with *I* > 2σ(*I*)
graphite	*R*_int_ = 0.019
ω scans	θ_max_ = 28.4°, θ_min_ = 3.2°
Absorption correction: multi-scan (*SADABS*; Bruker, 2007)	*h* = −11→16
*T*_min_ = 0.856, *T*_max_ = 0.897	*k* = −24→23
4996 measured reflections	*l* = −11→6

### Refinement

**Table:**

Refinement on *F*^2^	Secondary atom site location: difference Fourier map
Least-squares matrix: full	Hydrogen site location: inferred from neighbouring sites
*R*[*F*^2^ > 2σ(*F*^2^)] = 0.028	H atoms treated by a mixture of independent and constrained refinement
*wR*(*F*^2^) = 0.078	*w* = 1/[σ^2^(*F*_o_^2^) + (0.0475*P*)^2^ + 0.2164*P*] where *P* = (*F*_o_^2^ + 2*F*_c_^2^)/3
*S* = 1.07	(Δ/σ)_max_ < 0.001
1625 reflections	Δρ_max_ = 0.21 e Å^−^^3^
119 parameters	Δρ_min_ = −0.20 e Å^−^^3^
1 restraint	Absolute structure: Flack (1983), 372 Friedel pairs
Primary atom site location: structure-invariant direct methods	Flack parameter: 0.12 (11)

### Special details

**Table:**

Geometry. All e.s.d.'s (except the e.s.d. in the dihedral angle between two l.s. planes) are estimated using the full covariance matrix. The cell e.s.d.'s are taken into account individually in the estimation of e.s.d.'s in distances, angles and torsion angles; correlations between e.s.d.'s in cell parameters are only used when they are defined by crystal symmetry. An approximate (isotropic) treatment of cell e.s.d.'s is used for estimating e.s.d.'s involving l.s. planes.
Refinement. Refinement of *F*^2^ against ALL reflections. The weighted *R*-factor *wR* and goodness of fit *S* are based on *F*^2^, conventional *R*-factors *R* are based on *F*, with *F* set to zero for negative *F*^2^. The threshold expression of *F*^2^ > σ(*F*^2^) is used only for calculating *R*-factors(gt) *etc*. and is not relevant to the choice of reflections for refinement. *R*-factors based on *F*^2^ are statistically about twice as large as those based on *F*, and *R*- factors based on ALL data will be even larger.

### Fractional atomic coordinates and isotropic or equivalent isotropic displacement parameters (Å^2^)

**Table:**

	*x*	*y*	*z*	*U*_iso_*/*U*_eq_	
C1	0.65494 (18)	0.85229 (12)	0.6032 (3)	0.0505 (5)	
C2	0.7458 (2)	0.80891 (11)	0.5798 (3)	0.0568 (6)	
H2	0.8048	0.8285	0.5251	0.068*	
C3	0.75132 (18)	0.73752 (11)	0.6352 (3)	0.0510 (5)	
H3	0.8135	0.7094	0.6189	0.061*	
C4	0.66317 (14)	0.70786 (10)	0.7158 (3)	0.0384 (4)	
C5	0.57086 (16)	0.75001 (10)	0.7395 (3)	0.0455 (5)	
H5	0.5114	0.7303	0.7931	0.055*	
C6	0.56785 (17)	0.82139 (11)	0.6830 (3)	0.0500 (5)	
H6	0.5056	0.8496	0.6988	0.060*	
C7	0.6512 (2)	0.93096 (14)	0.5432 (4)	0.0782 (9)	
H7A	0.6955	0.9354	0.4495	0.117*	
H7B	0.6780	0.9637	0.6244	0.117*	
H7C	0.5776	0.9439	0.5175	0.117*	
C8	0.52239 (18)	0.56829 (12)	0.5701 (3)	0.0480 (5)	
H8A	0.5160	0.6123	0.5044	0.058*	
H8B	0.4701	0.5718	0.6562	0.058*	
C9	0.5000	0.5000	0.4707 (4)	0.0535 (8)	
H9	0.4379	0.5094	0.4025	0.064*	
S1	0.66864 (4)	0.61578 (2)	0.78225 (9)	0.04287 (14)	
O1	0.59049 (15)	0.60730 (8)	0.9065 (2)	0.0615 (4)	
O2	0.77949 (13)	0.59791 (8)	0.8137 (2)	0.0610 (5)	
N1	0.63280 (15)	0.56284 (9)	0.6355 (2)	0.0414 (4)	
H1	0.6771 (18)	0.5633 (14)	0.563 (3)	0.050*	

### Atomic displacement parameters (Å^2^)

**Table:**

	*U*^11^	*U*^22^	*U*^33^	*U*^12^	*U*^13^	*U*^23^
C1	0.0603 (13)	0.0396 (11)	0.0515 (13)	−0.0102 (9)	−0.0112 (10)	0.0031 (10)
C2	0.0545 (12)	0.0483 (11)	0.0675 (15)	−0.0129 (10)	0.0095 (13)	0.0028 (10)
C3	0.0442 (11)	0.0476 (10)	0.0613 (14)	−0.0010 (9)	0.0072 (11)	−0.0025 (10)
C4	0.0426 (11)	0.0346 (8)	0.0380 (9)	−0.0006 (7)	−0.0039 (9)	−0.0005 (7)
C5	0.0437 (10)	0.0421 (10)	0.0506 (13)	0.0009 (8)	0.0023 (9)	0.0025 (8)
C6	0.0481 (12)	0.0426 (10)	0.0594 (14)	0.0052 (9)	−0.0083 (10)	−0.0010 (9)
C7	0.094 (2)	0.0443 (13)	0.097 (2)	−0.0174 (12)	−0.0219 (18)	0.0166 (14)
C8	0.0488 (12)	0.0433 (10)	0.0519 (12)	−0.0037 (8)	−0.0063 (10)	0.0068 (9)
C9	0.0573 (19)	0.065 (2)	0.0387 (14)	−0.0183 (15)	0.000	0.000
S1	0.0551 (3)	0.0375 (2)	0.0360 (2)	0.00355 (18)	−0.0046 (3)	0.0038 (2)
O1	0.0904 (12)	0.0516 (9)	0.0425 (8)	−0.0022 (8)	0.0158 (9)	0.0068 (7)
O2	0.0633 (10)	0.0568 (9)	0.0627 (13)	0.0121 (7)	−0.0238 (9)	0.0014 (8)
N1	0.0462 (10)	0.0378 (8)	0.0403 (9)	0.0000 (7)	0.0016 (8)	−0.0001 (7)

### Geometric parameters (Å, °)

**Table:**

C1—C2	1.380 (3)	C7—H7B	0.9600
C1—C6	1.382 (3)	C7—H7C	0.9600
C1—C7	1.509 (3)	C8—N1	1.470 (3)
C2—C3	1.373 (3)	C8—C9	1.515 (3)
C2—H2	0.9300	C8—H8A	0.9700
C3—C4	1.387 (3)	C8—H8B	0.9700
C3—H3	0.9300	C9—C8^i^	1.515 (3)
C4—C5	1.383 (2)	C9—H9	0.9700
C4—S1	1.7567 (19)	S1—O1	1.4265 (17)
C5—C6	1.375 (3)	S1—O2	1.4276 (16)
C5—H5	0.9300	S1—N1	1.6199 (18)
C6—H6	0.9300	N1—H1	0.82 (3)
C7—H7A	0.9600		
			
C2—C1—C6	117.9 (2)	C1—C7—H7C	109.5
C2—C1—C7	120.9 (2)	H7A—C7—H7C	109.5
C6—C1—C7	121.2 (2)	H7B—C7—H7C	109.5
C3—C2—C1	121.8 (2)	N1—C8—C9	108.60 (16)
C3—C2—H2	119.1	N1—C8—H8A	110.0
C1—C2—H2	119.1	C9—C8—H8A	110.0
C2—C3—C4	119.3 (2)	N1—C8—H8B	110.0
C2—C3—H3	120.4	C9—C8—H8B	110.0
C4—C3—H3	120.4	H8A—C8—H8B	108.4
C5—C4—C3	120.04 (19)	C8—C9—C8^i^	113.3 (3)
C5—C4—S1	120.55 (14)	C8—C9—H9	109.0
C3—C4—S1	119.40 (15)	C8^i^—C9—H9	108.8
C6—C5—C4	119.33 (19)	O1—S1—O2	119.10 (12)
C6—C5—H5	120.3	O1—S1—N1	107.88 (10)
C4—C5—H5	120.3	O2—S1—N1	105.50 (10)
C5—C6—C1	121.67 (19)	O1—S1—C4	107.90 (9)
C5—C6—H6	119.2	O2—S1—C4	108.04 (9)
C1—C6—H6	119.2	N1—S1—C4	107.97 (10)
C1—C7—H7A	109.5	C8—N1—S1	119.73 (14)
C1—C7—H7B	109.5	C8—N1—H1	109.8 (17)
H7A—C7—H7B	109.5	S1—N1—H1	112.2 (18)
			
C6—C1—C2—C3	−0.9 (4)	C5—C4—S1—O1	−22.6 (2)
C7—C1—C2—C3	179.2 (3)	C3—C4—S1—O1	159.14 (18)
C1—C2—C3—C4	0.5 (4)	C5—C4—S1—O2	−152.60 (18)
C2—C3—C4—C5	0.1 (3)	C3—C4—S1—O2	29.1 (2)
C2—C3—C4—S1	178.35 (19)	C5—C4—S1—N1	93.76 (19)
C3—C4—C5—C6	−0.3 (3)	C3—C4—S1—N1	−84.50 (19)
S1—C4—C5—C6	−178.50 (17)	C9—C8—N1—S1	−163.87 (15)
C4—C5—C6—C1	−0.2 (3)	O1—S1—N1—C8	53.02 (18)
C2—C1—C6—C5	0.7 (4)	O2—S1—N1—C8	−178.68 (15)
C7—C1—C6—C5	−179.4 (2)	C4—S1—N1—C8	−63.35 (17)
N1—C8—C9—C8^i^	75.53 (14)		

Symmetry codes: (i) −*x*+1, −*y*+1, *z*.

### Hydrogen-bond geometry (Å, °)

**Table:**

*D*—H···*A*	*D*—H	H···*A*	*D*···*A*	*D*—H···*A*
N1—H1···O2^ii^	0.82 (3)	2.24 (3)	2.974 (2)	149 (2)
C7—H7C···O1^iii^	0.96	2.45	3.264 (3)	142

Symmetry codes: (ii) −*x*+3/2, *y*, *z*−1/2; (iii) −*x*+1, −*y*+3/2, *z*−1/2.
